# Ductus Venosus Agenesis in Fetuses: Epidemiological Data, Prenatal Findings, and Perinatal Outcomes—A Systematic Review

**DOI:** 10.3390/jcm15114174

**Published:** 2026-05-28

**Authors:** Radosław Karaś, Agata Michalczyk, Agata Włoch

**Affiliations:** 1 Department of Gynaecology and Obstetrics, Faculty of Health Sciences in Katowice, Medical University of Silesia, Wincentego Lipa 2 Street, 41-703 Ruda Śląska, Poland; awloch@sum.edu.pl; 2 Department of Congenital Heart Disease and Paediatric Cardiology, Silesian Centre for Heart Diseases in Zabrze, Marii Curie Skłodowskiej 9 Street, 41-800 Zabrze, Poland; agat.k.michalczyk@gmail.com

**Keywords:** agenesis of the ductus venosus, ductus venosus, congenital heart defects, fetal malformations, prenatal diagnosis, fetal circulation

## Abstract

**Background**: The fetal circulatory system is characterized by the presence of physiological vascular shunts—the ductus venosus, foramen ovale, and ductus arteriosus—which enable a significant portion of blood to bypass the pulmonary circulation and partially the hepatic portal system. This mechanism ensures preferential delivery of oxygenated blood to the brain, heart, and upper body. Agenesis of the ductus venosus (ADV) is a rare vascular anomaly associated with diverse anatomical variations and clinical outcomes. **Objectives:** This systematic review aimed to determine the prevalence of ADV and to assess the frequency of intrahepatic and extrahepatic types. Additional objectives were to identify the most common drainage sites of the umbilical vein (UV) in extrahepatic ADV, evaluate the genetic abnormalities, congenital heart defects, and extracardiac anomalies most frequently associated with ADV, and establish the prognosis of affected fetuses. **Methods**: A comprehensive literature search was conducted in the PubMed, Embase, and Web of Science databases using predefined and precise inclusion criteria. **Results**: The overall prevalence of ADV in the general population was 0.05%. The intrahepatic and extrahepatic types occurred with comparable frequencies, accounting for 51.2% and 48.8% of cases, respectively. In extrahepatic ADV, the most common drainage site of the UV was the right atrium (48%). The most frequent genetic abnormalities were trisomy 21 (12%) and Turner syndrome (6%). Among congenital heart defects, ventricular septal defect (22.7%) and atrioventricular septal defect (8.8%) were most prevalent. Functional consequences were observed in 37.4% of fetuses with isolated ADV, most commonly cardiomegaly. **Conclusions**: ADV is a rare fetal vascular anomaly. With the increasing availability and utilization of prenatal diagnostic techniques, expanding knowledge of this condition and its clinical implications is essential for accurate diagnosis, management, and prognostic assessment.

## 1. Introduction

The fetal circulatory system is characterized by a unique anatomy and physiology. Its distinguishing feature is that gas exchange occurs in the placenta rather than in the lungs. Consequently, a significant portion of fetal blood bypasses the lungs and, to some extent, the liver due to the presence of physiological vascular shunts: the ductus venosus (DV), the foramen ovale (FO), and the ductus arteriosus (DA, ductus Botalli).

Oxygenated blood returns from the placenta to the fetus via a single umbilical vein (UV). Approximately 50% of this volume passes through the DV, bypassing the hepatic portal circulation, then joins the inferior vena cava (IVC) and reaches the right atrium (RA) [1]. The Eustachian valve directs the stream of oxygen-rich blood through the FO into the left atrium (LA), thereby bypassing the pulmonary circulation [1,2]. Subsequently, blood flows through the mitral valve into the left ventricle, from where it is ejected into the ascending aorta.

The remaining blood in the RA, which is not shunted through the FO, passes through the tricuspid valve into the right ventricle and is then pumped into the pulmonary trunk during systole [1]. The right ventricle receives predominantly blood originating from the superior vena cava (SVC) [2]. Due to the presence of the DA and elevated pulmonary vascular resistance (PVR), only 10–20% of the right ventricular output passes through the pulmonary circulation, while the majority is diverted via the DA into the descending aorta [3].

**Figure 1:**
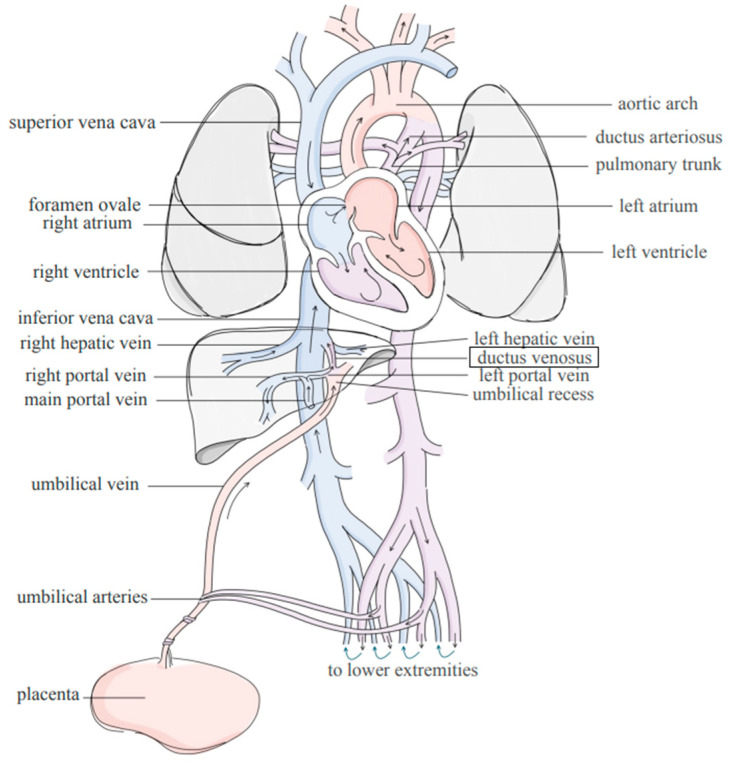
Schematic representation of the fetal circulatory system.

This arrangement of the fetal circulation ensures preferential delivery of oxygenated blood to the brain, heart, and upper body [1,3]. A schematic representation of the fetal circulation is shown in Figure 1. In the second trimester of pregnancy, the mean length of the DV is 0.68 cm and its diameter is 0.4 cm, whereas in the third trimester these values increase to 1.76 cm and 0.46 cm, respectively [4]. After birth, the DV transforms into the ligamentum venosum [5].

**Figure 2:**
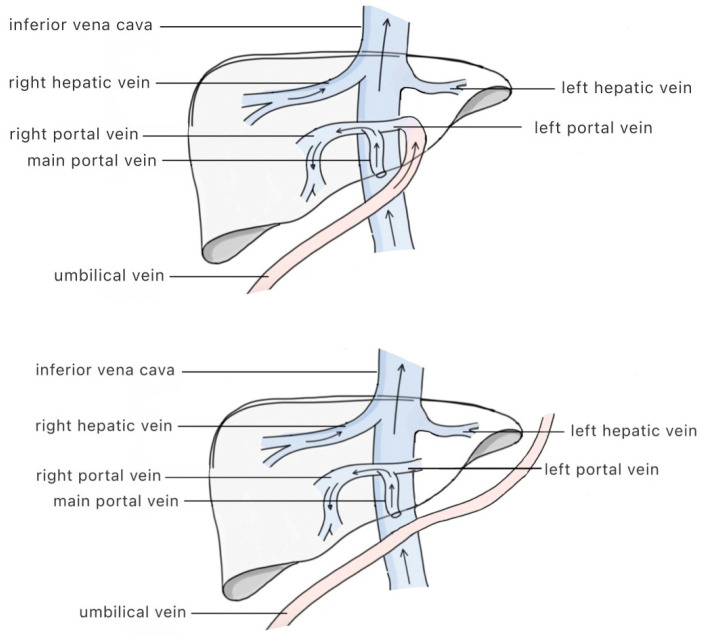
Schematic representation of the intrahepatic type of ADV (**top**) and the extrahepatic type of ADV (**bottom**).

Agenesis of the ductus venosus (ADV) was first described by Paltauf R. in 1888 [6]. It is a very rare anomaly, with an estimated incidence of <1% [7,8,9,10]. ADV may occur in either an intrahepatic or extrahepatic variant. The intrahepatic variant is characterized by drainage of the UV into the portal sinus or hepatic veins in the absence of the DV. In turn, the extrahepatic variant refers to a situation in which the UV, bypassing the liver, drains into the right atrium, the inferior vena cava, the iliac vein, or the coronary sinus [7,11,12], and less commonly into other venous vessels. The intrahepatic and extrahepatic types of ADV are presented in Figure 2.

ADV may present as an isolated defect or as a non-isolated condition associated with other structural anomalies or genetic abnormalities. It is more frequently observed in fetuses with chromosomal abnormalities, including trisomy 21, trisomy 18, trisomy 13, and Turner syndrome [12]. The isolated form may still lead to functional disturbances in the fetus, such as cardiomegaly, subcutaneous edema, pleural effusion, and ascites [13]. Among the structural anomalies associated with ADV, congenital heart defects are commonly observed (including ventricular septal defect [VSD], atrioventricular septal defect [AVSD], double outlet right ventricle [DORV], and coarctation of the aorta [CoA]), as well as malformations involving other organ systems.

The aim of this systematic review was to determine the prevalence of ADV, including its distribution according to fetal sex. Additionally, the study sought to assess the frequency of intrahepatic and extrahepatic types of ADV, identify the most common drainage sites of the UV in cases of extrahepatic ADV, and evaluate the genetic abnormalities, congenital heart defects, and extracardiac anomalies most frequently associated with this condition. Furthermore, this review aimed to establish the prognosis of fetuses diagnosed with ADV.

Diagnosis of ADV in the fetus has important clinical implications. It necessitates detailed evaluation for structural anomalies, including cardiac defects, and, in some cases, chromosomal abnormalities. Depending on the phenotype and associated abnormalities, ADV may be associated with a highly variable prognosis—ranging from isolated cases with a relatively favorable outcome to conditions carrying a high risk of fetal circulatory failure and intrauterine fetal demise. Early diagnosis therefore enables appropriate pregnancy surveillance and planning of further genetic and cardiac assessment. Depending on the associated abnormalities, referral of the patient to a suitably equipped tertiary referral center may be indicated. Accurate diagnosis of ADV and coexisting anomalies allows for optimization of perinatal management.

## 2. Materials and Methods

### 2.1. Data Source

The literature review was designed and conducted in accordance with the PRISMA statement [14] to ensure transparent, comprehensive, and unbiased reporting of data. The study was not registered. PRISMA Checklist and PRISMA abstract checklist are listed as Supplementary Materials. Electronic databases, including PubMed, Embase, and Web of Science, were systematically searched using relevant terms and keywords derived from the Medical Subject Headings (MeSH) list: ductus venosus, agenesis, absent, and absence. The database search was carried out between March and September 2025.

### 2.2. Inclusion and Exclusion Criteria

Prospective and retrospective studies were included, as well as—given the rarity of ADV—case reports and case series. Studies describing ADV were considered regardless of the trimester at which the anomaly was detected. Both singleton and multiple pregnancies were included.

The exclusion criteria comprised: systematic reviews, conference abstracts, letters to the editor, publications in languages other than English, studies for which full-text access was unavailable, studies concerning ADV in animals, and studies describing ADV exclusively in neonates.

### 2.3. Study Selection

**Figure 3:**
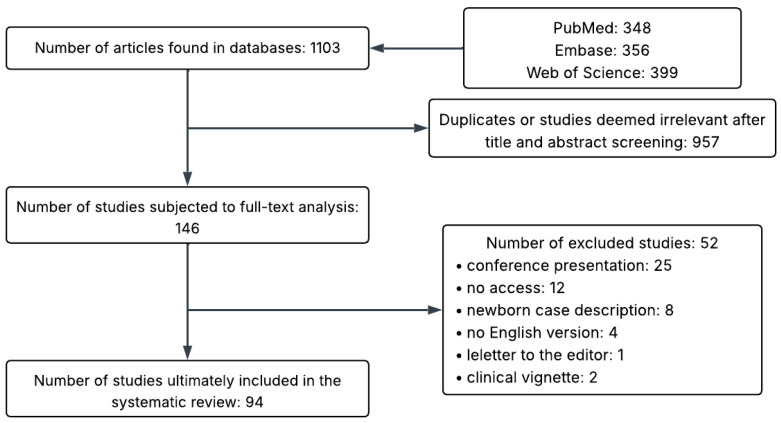
Flow diagram of the identification of studies included in the systematic review.

Initially, the titles and abstracts of the identified articles were screened. Subsequently, full-text analyses were performed for studies deemed potentially relevant to the systematic review. All records retrieved through the electronic search were organized alphabetically, and duplicates were removed. The study search and selection process is illustrated in Figure 3.

**Figure 4:**
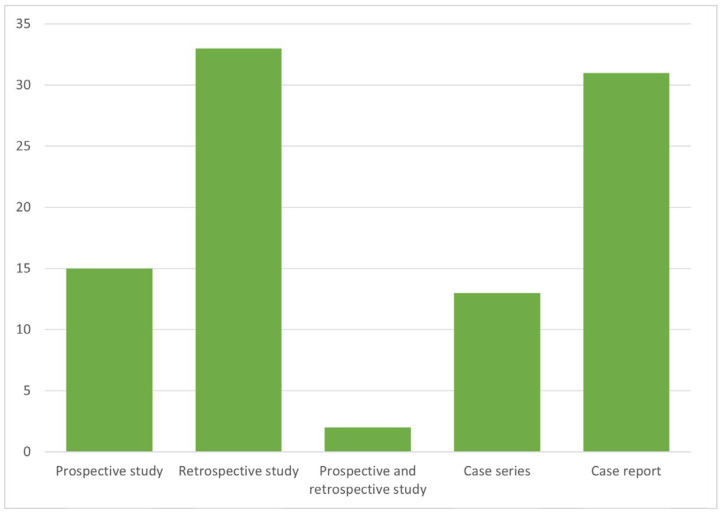
Number of included studies according to study type.

**Figure 5:**
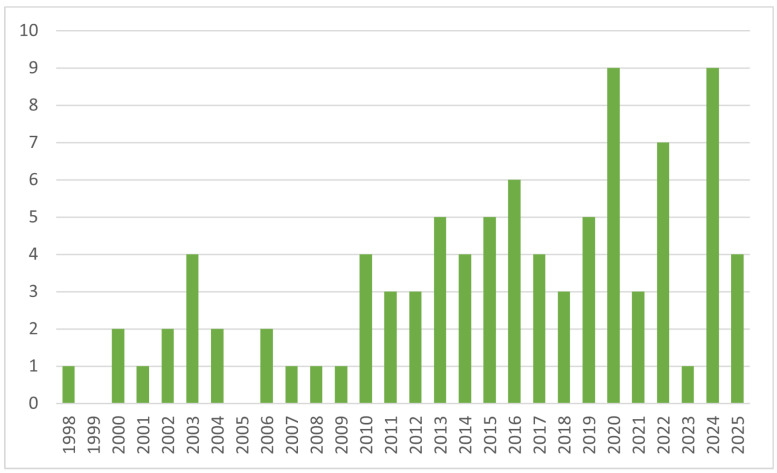
Number of included studies according to year of publication.

The literature search and initial screening of publications were conducted by the lead author under continuous and active academic supervision from the remaining co-authors. Any discrepancies or uncertainties were resolved through discussion among the authors. Ultimately, 94 scientific publications [7,8,9,10,11,12,13,15,16,17,18,19,20,21,22,23,24,25,26,27,28,29,30,31,32,33,34,35,36,37,38,39,40,41,42,43,44,45,46,47,48,49,50,51,52,53,54,55,56,57,58,59,60,61,62,63,64,65,66,67,68,69,70,71,72,73,74,75,76,77,78,79,80,81,82,83,84,85,86,87,88,89,90,91,92,93,94,95,96,97,98,99,100,101] were included in the review. The distribution of included studies by type is presented in Figure 4, while the number of included studies according to year of publication is shown in Figure 5.

The following data were collected from the included publications: year of publication, study design, incidence of ADV, frequency of intrahepatic and extrahepatic types, prevalence of isolated versus non-isolated ADV, associated extracardiac anomalies, associated congenital heart defects, karyotype, sex, site of UV drainage in cases of the extrahepatic type, and pregnancy outcome.

Both prenatally diagnosed anomalies and those identified postnatally or at autopsy were included in the assessment of extracardiac and cardiac defects. The isolated form was defined as the presence of ADV alone, as well as cases with ultrasonographic findings that may represent a primary or secondary functional consequence of ADV.

Case reports and case series were excluded from the analysis of the prevalence of isolated and non-isolated forms due to their inherent bias toward reporting ADV cases associated with additional anomalies. Only studies reporting both the number of ADV cases and the total screened population were included in the incidence analysis. Analyses addressing anatomical subtype, associated anomalies, and clinical outcomes additionally incorporated case series, case reports, and descriptive studies lacking denominator populations.

## 3. Results

In studies eligible for incidence estimation, ADV was identified in 272 fetuses among 222 561 screened pregnancies, corresponding to an overall incidence of 0.12%. When the analysis was restricted to studies involving unselected general screening populations (i.e., excluding pregnancies referred for targeted prenatal assessment), the incidence decreased to 0.05%. The incidence rates reported across individual studies and study populations are summarized in Table 1.

Among the 142 cases in which fetal sex was determined, ADV occurred with a similar frequency in male and female fetuses. Specifically, 77 out of 142 cases (54.2%) involved male fetuses, while 65 out of 142 cases (45.8%) involved female fetuses.

**Figure 6:**
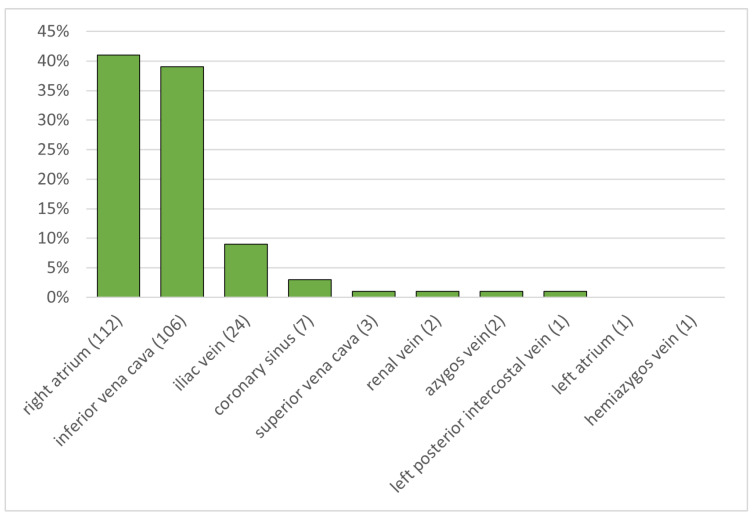
Proportion of drainage sites of the umbilical vein in fetuses with extrahepatic agenesis of the ductus venosus.

The intrahepatic variant of ADV was identified in 315 (51%) fetuses, whereas the extrahepatic variant was observed in 300 (49%) cases. The most common sites of UV drainage in the extrahepatic type of ADV are the right atrium (41%) and the inferior vena cava (39%). Detailed information on UV drainage sites is presented in Figure 6.

Among the analyzed cases of fetuses with ADV, 350 underwent genetic testing. A normal karyotype was observed in 224 (64%) fetuses, whereas 126 cases (36%) exhibited abnormal genetic results. Among the abnormal findings, the most frequently identified conditions were trisomy 21 (33.3%) and Turner syndrome (16.7%). Less common were trisomy 18 (7.9%), Noonan syndrome (7.14%), and trisomy 13 (13.2%).

Additionally, two cases each were reported for 45,X0/46,XY, 22q11.2 microdeletion, 10p deletion, and mosaic 47,XY,+mar(15)/46,XY(15). Numerous genetic abnormalities were observed in only a sin-gle case each, including: Caffey syndrome, 22q17 deletion, Adams–Oliver syndrome, Angelman syndrome, MICPCH syndrome, PHACE syndrome, RA-SA1-related disorder, trisomy 22, trisomy 9, chromosome 5 microdeletion, achondroplasia, Beckwith–Wiedemann syndrome, 46,X,der(X), 46,XX,add(5)(p15), Robertsonian translocation, mosaic tetraploid 10% on placental biopsy, 22q deletion, chromosome 2 mosaicism, cranioectodermal dysplasia 2 (WDR35 mutation), 13p deletion, 2p12 deletion, 4p deletion (Wolf–Hirschhorn syndrome), placental trisomy 22, Smith–Lemli–Opitz syndrome, X-linked Opitz syndrome, Pallister–Killian syndrome, Jacobsen syndrome, Der(8), 46,XY,dup(8)(q21.1q22)/47,idem,+r(8), 46,XY,inv(9)(p12q13), 15q11.2-q13.1 microdeletion, 16q24.3 microdeletion, rsa(X,13,18,21)x2, 6p25.3p22.3 duplication located on chromosome 22p11.2, and translocation 46,XX,add(12)(q24).

The prevalence of ADV according to the type of genetic abnormality is presented in Table 2.

The isolated form of ADV was observed in 241 cases (45%), whereas the non-isolated form was identified in 295 cases (55%).

ADV accounts for 10% of fetal venous anomalies (6/61). Among venous anomalies, persistent left superior vena cava (PLSVC) is observed significantly more frequently (57%), while similar frequencies are reported for umbilical vein varix (10%), total anomalous pulmonary venous connection (TAPVC, 7.5%), and interrupted IVC (7.5%) [30]. The prevalence of ADV in various abnormalities is presented in Table 3.

A total of 646 fetuses were analyzed for the presence of associated structural and functional anomalies. The concomitant abnormalities of the genitourinary system, musculoskeletal system, central nervous system, gastrointestinal system, and other anomalies associated with ADV are presented in Table 4. Venous system anomalies coexisting with ADV are summarized in Table 5.

The most common feature of cardiovascular dysfunction in fetuses with ADV is cardiomegaly. Table 6 presents the primary and secondary features of cardiovascular compromise in fetuses with ADV.

Among 251 cases of structural heart defects identified in fetuses with ADV, the most common anomalies were ventricular septal defect (VSD, 22.7%), atrioventricular septal defect (AVSD, 8.8%), double outlet right ventricle (DORV, 6%), and coarctation of the aorta (5.9%). Table 7 presents the structural anomalies of the heart, great vessels, and cardiac valves coexisting in fetuses with ADV.

Additionally, single cases were reported for: hyperechogenic foci in the heart (7), hypertrophic cardiomyopathy (4), bradycardia (3), tachycardia (1), contractility disorders (2), first-degree atrioventricular block (1), third-degree atrioventricular block (1), heart failure (2), restrictive foramen ovale (1), juxtaposition of IVC/aorta (1), atrial septal aneurysm (1), and floppy or slightly thickened atrioventricular valves (1).

**Figure 7:**
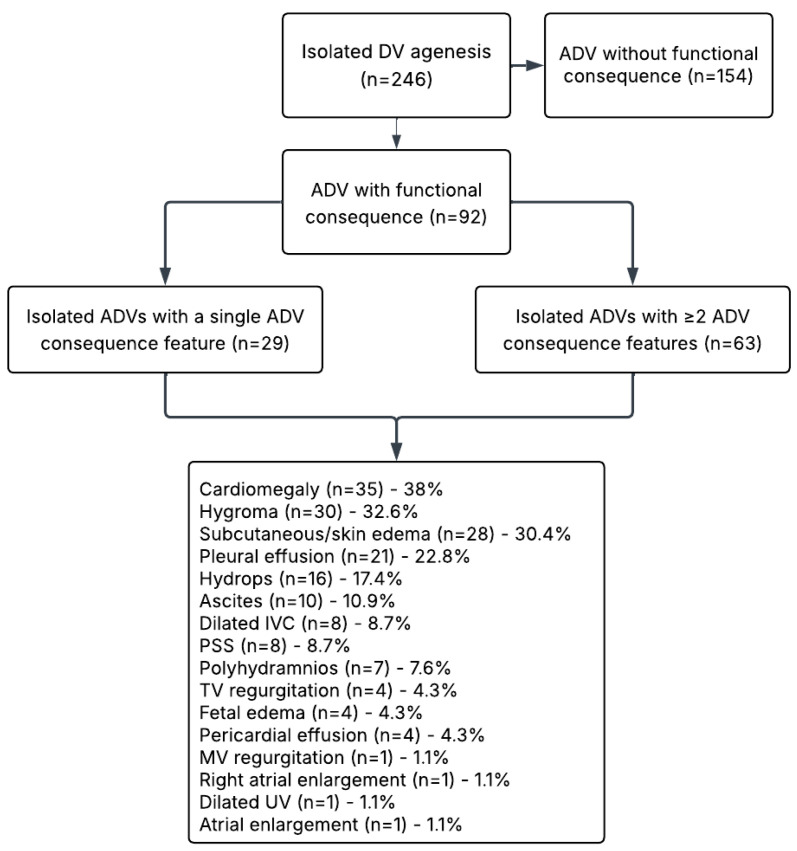
The prevalence of isolated agenesis of the ductus venosus without functional complications and with functional complications, and the prevalence of features resulting from isolated agenesis of the ductus venosus. IVC—inferior vena cava, PSS—portosystemic shunt, TV—tricuspid valve, MV—mitral valve, UV—umbilical vein.

A total of 244 fetuses were analyzed for features that could represent functional consequences of isolated ADV. Among these, 62.6% showed no functional consequences. In fetuses exhibiting functional complications of ADV (37.4%), the most common findings were cardiomegaly, subcutaneous/skin edema, and pleural effusion. Details are presented in Figure 7.

**Figure 8:**
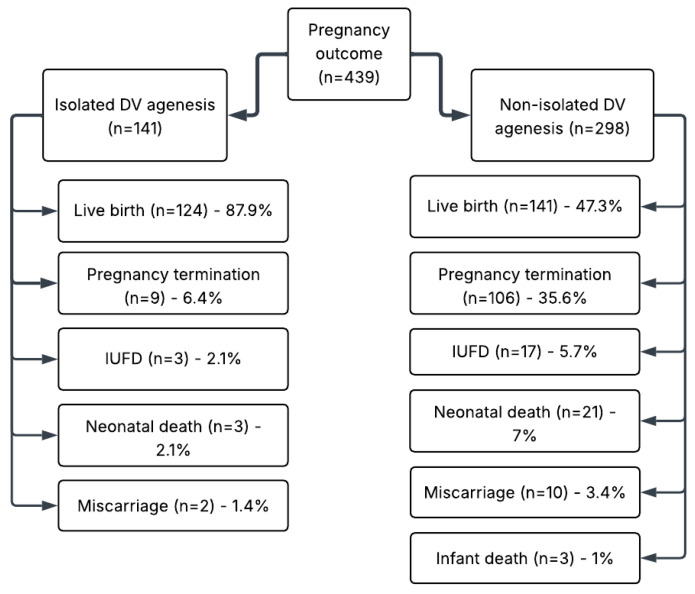
Pregnancy outcomes according to agenesis of the ductus venosus type (isolated vs. non-isolated). IUFD—intrauterine fetal death.

The isolated form of ADV resulted in live birth in 87.9% of cases. Miscarriage, intrauterine fetal death, and neonatal death were reported in single cases. In contrast, the non-isolated form of ADV led to live births in 47.3% of cases, with pregnancy termination being significantly more frequent in this group. Detailed information is presented in Figure 8.

## 4. Conclusions

Agenesis of the ductus venosus (ADV) is a rare congenital vascular anomaly in the fetus. It is frequently associated with chromosomal abnormalities, structural and functional cardiac defects, and extracardiac malformations, all of which significantly influence clinical outcomes and prognosis. With the increasing availability of prenatal ultrasound and fetal echocardiography, awareness and understanding of ADV continue to improve.

Future research should focus on further elucidating the relationship between ADV and the coexistence of other structural anomalies, including congenital heart defects, as well as genetic abnormalities. Studies are also needed to more precisely define cases requiring intensified surveillance or referral to tertiary care centers, as well as to provide a comprehensive long-term assessment of postnatal outcomes in fetuses with ADV.

## Figures and Tables

**Table 1:** Prevalence of agenesis of the ductus venosus in populations from different countries.

Number of Fetuses Studied	Number of ADV Cases (%)	Country	Authors
7552	12 (1.6%)	India	Bakhru S et al. [11]
8304	13 (0.16%)	Greece	Souka A et al. [15]
11,491	10 (0.09%)	Spain	Garcia-Delgado R et al. [7]
5810	35 (0.6%)	Poland	Wiechec M et al. [16]
65,840	26 (0.04%)	United Kingdom	Staboulidou I et al. [8]
990	6 (0.6%)	USA	Acherman RJ et al. [17]
3517	18 (0.5%)	Romania	Nagy RD et al. [18]
25,155	3 (0.01%)	China	Torcia E et al. [9]
1499	1 (0.07%)	India	Karmegaraj B et al. [19]
2610	4 (0.2%)	Japan	Takita H et al. [20]
6114	11 (0.2%)	Romania, Greece	Iliescu DG et al. [10]
174	2 (1.2%)	Israel	Sciaky-Tamir Y et al. [21]
310	4 (1.3%)	USA	Kalache K et al. [22]
18,500	14 (0.08%)	China	Ozsurmeli M et al. [23]
13,101	15 (0.1%)	Israel	Yagel S et al. [24]
3691	3 (0.1%)	India	Thomas A et al. [25]
7048	3 (0.04%)	Taiwan	Tseng WH et al. [26]
14,793	25 (0.2%)	Romania	Nagy RD et al. [27]
6133	61 (1%)	Poland	Rajs B et al. [28]
19,929	6 (0.03%)	India	Chaudhary YS et al. [29]

**Table 2:** Prevalence of agenesis of the ductus venosus according to genetic abnormality.

Chromosomal Anomalies	Number of Cases	Number of ADV Cases	Authors
Trisomy 13	56	2 (3.6%)	Rajs B et al. [28]
Turner syndrome	72	7 (9.7%)	Wiechec M et al. [30] and Staboulidou I et al. [8]
Trisomy 21	334	2 (0.6%)	Staboulidou I et al. [8]
Noonan syndrome	13	5 (38.5%)	Tangshewinsirikul C et al. [31]
Trisomy 18	113	1 (0.9%)	Staboulidou I et al. [8]

**Table 3:** Prevalence of agenesis of the ductus venosus in various anatomical anomalies.

Abnormality	Number of Cases	Number of ADV Cases	Authors
Persistent left superior vena cava	229	2 (0.9%)	Minsart AF et al. [32]
Persistent right umbilical vein	23	2 (8.7%)	Kumar SV et al. [33]
Single umbilical artery	261	1 (0.4%)	Araujo Júnior E et al. [34]
Agenesis or dysgenesis corpus callosum	43	2 (4.7%)	Rodriguez MA et al. [35]
Tricuspid atresia	65	1 (1.5%)	Berg C et al. [36]

**Table 4:** Associated structural and functional anomalies in fetuses with agenesis of the ductus venosus (*n* = 646).

Anomaly Associated with ADV	Number	Anomaly Associated with ADV	Number
Genitourinary system anomalies		Central nervous system (CNS) anomalies	
Pyelectasis	13	Cerebellar vermis agenesis/hypoplasia/Dandy–Walker malformation/cerebellar hypoplasia	15
Renal agenesis	4	Ventriculomegaly	9
Horseshoe kidney	4	Corpus callosum agenesis/hypoplasia	7
Hydronephrosis	3	Microcephaly	7
Micropenis	3	Holoprosencephaly	6
Pelvic kidney	2	Choroid plexus cysts	3
Echogenic kidneys	2	Brachycephaly	1
Hypospadias	2	Cranial skin edema	1
Absent bladder	2	Dolichocephaly	1
Megalourethra	1	Enlarged fourth ventricle	1
Renal dysplasia	1	Exencephaly	1
Duplication of left renal artery	1	Plagiocephaly	1
Extrophy of bladder	1	Hippocampal abnormalities	1
Megacystis	1	Large dorsal cyst in fetal brain	1
Ambiguous genitalia	1	Hydranencephaly	1
Gonadal dysgenesis	1	Blake’s pouch cyst	1
Renal cysts	1	Spina bifida	1
Hypoplastic scrotum	1	intracranial cystic lesions	1
Hydroureter	1	Hydrocephalus	1
Cryptorchidism	1	Enlarged cisterna magna	1
Genitals abnormalities	1	Enlargement of the Sylvian fissure	1
Bifid scrotum	1	Occipital encephalocele	1
Musculoskeletal anomalies		Gastrointestinal system anomalies	
Abnormal/short/absent long bones	15	Esophageal atresia	9
Clubfoot	8	Duodenal atresia	8
Spinal deformities	4	Anal atresia	7
Kyphoscoliosis	3	Hepatic calcifications/hyperechogenic foci in the liver	5
Abnormal hands and/or feet	3	Hyperechogenic bowel	5
Clenched hands	3	Diaphragmatic hernia	5
Polydactyly	2	Small stomach	5
Rocker bottom feet	2	Hypoplasia of the left hepatic lobe segment	3
Syndactyly	2	Gallbladder agenesis	3
Camptodactyly	1	Intestinal malrotation	3
Distorted feet	1	Anorectal malformation	2
Arthrogryposis	1	Rectovestibular fistula	1
Hemivertebrae	1	Right-sided stomach	1
Pectus excavatum	1	Gastroschisis	1
Bell-shaped thorax	1	Intestinal obstruction	1
Overlapping fingers	1	Craniofacial anomalies	
Lumbar hemivertebrae	1	Nasal bone hypoplasia/absence	8
Respiratory system anomalies		Cleft lip and/or palate	8
Lung aplasia/hypoplasia	3	Hypertelorism	7
Tracheoesophageal fistula	2	Micrognathia	4
Persistent pulmonary hypertension of the newborn	1	Microphthalmia	4
Tracheal atresia	1	Low-set ears	3
Other anomalies		Retrognathia	3
FGR/IUGR	42	Choanal hypoplasia	3
Polyhydramnios	27	Frontal bossing	2
Increased NT	20	Nasal bone maldevelopmen	2
Omphalocele	9	Flat face	1
Oligohydramnios	6	Hypoplastic nasal bridge	1
Heterotaxy syndrome	5	Beaked nose	1
Myopathy	3	Strawberry-shaped head	1
Increased nuchal fold	3	Cheilognathopalatoschisis	1
Aorto-ombilico-hepatic fistula	3	Broad nasal bridge	1
VECTREL association	3	Pierre Robin sequence	1
Lateral body wall defect/Limb-body wall complex (LBWC)	2	Coloboma	1
Axial hypotonia	2	Depressed cranial bones	1
Body-stalk syndrome	2	Increased prenasal thickness	1
Fetal hypertrophy	1	Flat nose root	1
Thoraco-abdominal schisis	1	Placental and umbilical cord abnormalities	
Hemangiomas of the skin	1	SUA	48
Micromelia	1	Umbilical vein varices	5
Increased inter-mamillary distance	1	Placentomegaly	3
Nuchal cysts	1	Hypercoiled umbilical cord	3
Asplenia	1	PRUV	2
Situs inversus	1	Umbilical cord cyst	1
Diaphragmatic eventration	1	Placental chorioangioma	1
Multiple hemangiomas	1	Endocrine system anomalies	
Miller-Lev-Paul syndrome	1	Thymic hypoplasia/agenesis	3
Severe chylothoraces	1	Congenital adrenal hyperplasia (CAH) without salt-wasting	1
Bilateral sensorineural deafness	1	Hypoparathyroidism	1
Severe antibody mediated anemia	1		

FGR—fetal growth restriction; IUGR—intrauterine growth restriction; NT—nuchal translucency; SUA—single umbilical artery; PRUV—persistent right umbilical vein. The table headers highlighted in gray indicate the organ systems to which the anatomical anomalies listed below belong.

**Table 5:** Venous system anomalies coexisting with agenesis of the ductus venosus (*n* = 646).

Venous System Anomalies	Number
Persistent left superior vena cava	25
Total portal venous system agenesis	12
Partial portal venous system agenesis	9
Congenital agenesis of the portal venous system: type I: 4 type Ib: 1 type IIa: 1	6
Interrupted inferior vena cava	6
Bilateral superior vena cava	5
Infradiaphragmatic partial anomalous pulmonary venous connection	1
No inferior vena cava	1
Rudimentary left cardinal vein	1

**Table 6:** Primary and secondary features of cardiovascular dysfunction in fetuses with agenesis of the ductus venosus (*n* = 646).

Anomaly Associated with ADV	Number (%)
Cardiomegaly	126 (19.5%)
Hygroma	61 (9.4%)
Subcutaneous/skin edema	58 (9%)
Portosystemic shunt	58 (9%)
Hydrops	52 (8%)
Pleural effusion	49 (7.6%)
Pericardial effusion	27 (4.2%)
Ascites	22 (3.4%)
Tricuspid valve regurgitation	21 (3.3%)
Dilated inferior vena cava	21 (3.3%)
Cystic hygroma	19 (2.9%)
Mitral valve regurgitation	7 (1.1%)
Dilated right atrium	7 (1.1%)
Dilated right ventricle	7 (1.1%)
Biventricular hypertrophy	7 (1.1%)
Dilated umbilical vein	4 (0.6%)
Dilated coronary sinus	4 (0.6%)
ventricular disproportion	4 (0.6%)
Nuchal edema	3 (0.5%)
Right ventricular hypertrophy	3 (0.5%)
Hepatomegaly	2 (0.3%)
Atrial enlargement	1 (0.15%)
Pulmonary valve regurgitation	1 (0.15%)
Iliac vein dilatation	1 (0.15%)

**Table 7:** Structural anomalies of the heart, valves, and great vessels in fetuses with agenesis of the ductus venosus.

Anomaly Associated with ADV	Number	Anomaly Associated with ADV	Number
Septal and cardiac chamber defects		Valvular defects	
Ventricular septal defect	57	Pulmonary stenosis	8
Atrioventricular septal defect	22	Pulmonary valve atresia	5
Atrial septal defect	14	Aortic stenosis	3
Single ventricle	10	Mitral valve atresia	3
Single atrium	6	Pulmonary valve dysplasia	3
Septal agenesis	1	Tricuspid valve atresia	1
Tri-atrium	1	Subaortic stenosis	2
Conotruncal heart defects		Bicuspid aortic valve	2
Double outlet right ventricle	15	Bicuspid pulmonary valve	1
Persistent truncus arteriosus	12	Aortic valve dysplasia	1
Transposition of the great arteries	5	Tricuspid valve dysplasia	1
Fallot syndrome	4	Common atrioventricular valve	1
Double outlet ventricle	1	Syndromic/complex defects	
Common arterial trunk from right ventricle	1	Hypoplastic left heart syndrome	8
Outflow tracts is parallel	1	Hypoplastic left ventricle	8
Aortic arch and great vessel defects		Dextrocardia	5
Coarctation of the aorta	15	Ebstein anomaly	4
Aberrant right subclavian artery	8	Atrial isomerism	2
Right aortic arch	6	Pulmonary trunk hypoplasia	1
Aortic arch hypoplasia	3	Discordant ventricles	1
Double aortic arch	2	Congenital cardiomyopathies	
Interrupted aortic arch	1	Left ventricular non-compaction	2
Bovine aortic arch	1		
Abnormal pulmonary venous return	3		

The gray color indicates the categories of structural heart defects.

## Data Availability

No new data were generated in this study. All data analyzed were obtained from previously published articles, which are cited in the reference list.

## Supplementary Materials

The following supporting information can be downloaded at: https://www.mdpi.com/article/10.3390/jcm15114174/s1, Supplementary files: PRISMA Checklist; PRISMA abstract checklist. Reference [102] is cited in the Supplementary Materials.

## Abbreviations

The following abbreviations are used in this manuscript:

DV	ductus venosus
FO	foramen ovale
DA	ductus arteriosus
UV	umbilical vein
IVC	inferior vena cava
RA	right atrium
LA	left atrium
SVC	superior vena cava
PVR	pulmonary vascular resistance
ADV	agenesis of ductus venosus
VSD	ventricular septal defect
AVSD	atrioventricular septal defect
DORV	double outlet right ventricle
CoA	coarctation of the aorta
MeSH	medical subject headings
PLSVC	persistent left superior vena cava
TAPVC	total anomalous pulmonary venous connection
PRUV	persistent right umbilical vein
SUA	single umbilical artery
CC	corpus callosum
CNS	central nervous system
FGR	fetal growth restriction
IUGR	intrauterine growth restriction
NT	nuchal translucency
LBWC	limb-body wall complex
CAH	congenital adrenal hyperplasia
PSS	portosystemic shunt
CS	coronary sinus
PTA	persistent truncus arteriosus
RAA	right aortic arch
TV	tricuspid valve
MV	mitral valve
IUFD	intrauterine fetal death
